# Comprehensive 3D phenotyping reveals continuous morphological variation across genetically diverse sorghum inflorescences

**DOI:** 10.1111/nph.16533

**Published:** 2020-04-16

**Authors:** Mao Li, Mon‐Ray Shao, Dan Zeng, Tao Ju, Elizabeth A. Kellogg, Christopher N. Topp

**Affiliations:** ^1^ Donald Danforth Plant Science Center St Louis MO 63132 USA; ^2^ Department of Computer Science and Engineering Washington University St Louis MO 63130 USA

**Keywords:** inflorescences, panicle, phenomics, phenotyping, sorghum, X‐ray

## Abstract

● Inflorescence architecture in plants is often complex and challenging to quantify, particularly for inflorescences of cereal grasses. Methods for capturing inflorescence architecture and for analyzing the resulting data are limited to a few easily captured parameters that may miss the rich underlying diversity.
● Here, we apply X‐ray computed tomography combined with detailed morphometrics, offering new imaging and computational tools to analyze three‐dimensional inflorescence architecture. To show the power of this approach, we focus on the panicles of *Sorghum bicolor*, which vary extensively in numbers, lengths, and angles of primary branches, as well as the three‐dimensional shape, size, and distribution of the seed.
● We imaged and comprehensively evaluated the panicle morphology of 55 sorghum accessions that represent the five botanical races in the most common classification system of the species, defined by genetic data. We used our data to determine the reliability of the morphological characters for assigning specimens to race and found that seed features were particularly informative.
● However, the extensive overlap between botanical races in multivariate trait space indicates that the phenotypic range of each group extends well beyond its overall genetic background, indicating unexpectedly weak correlation between morphology, genetic identity, and domestication history.

## Introduction

Plant organs show tremendous diversity in size and shape as a result of differential growth and patterning throughout development. Measurements of plant structures such as inflorescences or leaves for botany, crop breeding, or ecology studies most often use manual observation or the analysis of two‐dimensional (2D) images, the latter ranging from electron microscopy (e.g. Buzgo *et al.*, 2004; Vollbrecht *et al.*, 2005; Prenner & Rudall, 2007; Kellogg *et al.*, 2013) to high‐throughput camera imaging (Chen *et al.*, 2014; Gehan & Kellogg, 2017). More recently, imaging techniques have been developed that enable the reconstruction and analysis of plant tissue or structures in three dimensions. At the microscopic scale, three‐dimensional (3D) reconstructions of cells can be achieved using several techniques, such as confocal or scanning electron microscopy (Bougourd *et al.*, 2000; Denk & Horstmann, 2004). On the other hand, at field scales, plant height and other traits can be measured using 3D laser scanners or unmanned aerial systems (Friedli *et al.*, 2016; Malambo *et al.*, 2018), resulting in phenomics becoming an increasingly important tool in field research.

Between these two scales, specific structures of and within plants can be imaged in three dimensions using high‐resolution X‐ray computed tomography (XRT; Stuppy *et al.*, 2003; Dhondt *et al.*, 2010; Pérez‐Torres *et al.*, 2015; Rogers *et al.*, 2016; Jiang *et al.*, 2019). Although the processing and analysis of 3D images for complex biological structures remains nontrivial, automated computational tools providing precise analysis for 3D imaging are becoming increasingly powerful, permitting segmentation, reconstruction, registration, visualization, feature extraction, and modeling. For example, 3D wheat grains have been segmented and measured from images of the wheat panicle (Hughes *et al.*, 2017), and 3D branching topology has been characterized comprehensively in grapevine inflorescences (Li *et al.*, 2017, 2019). Furthermore, software such as Chimera (Pettersen *et al.*, 2004) has been used widely for the visualization and analysis of molecular structures, density maps, 3D microscopy, and associated data (Goddard *et al.*, 2017), and topological data analysis tools such as the medial axis (Blum, 1967) have been used extensively for analyzing shape structure and for forming shape descriptors. The medial axis, in particular, is a topological curve skeleton that is a complete shape descriptor, meaning that it can be used to reconstruct inflorescence shape; the geometry and thickness along the medial axis can then be used to quickly identify the main stalk and primary branches. Because the internal structures of grass inflorescences are often occluded in flat 2D scans, these shape analysis methods can only be fully utilized with 3D images.

Here, we apply these tools to a long‐standing problem of morphological diversity and variation within *Sorghum bicolor*. The inflorescences of sorghum are referred to as panicles, with a central monopodial axis and lateral branches that then further branch (Bell, 2008). Spikelets on the branches occur in pairs, with one hermaphrodite and one sterile, and in the hermaphrodite spikelet is one fertile and one sterile floret. The terminal spikelet on each branch is morphologically similar to the sterile spikelet from the immediately proximal pair, giving the appearance of a triplet of spikelets. From a mature ovary, the grain that develops is a type of indehiscent fruit known as a caryopsis, where the pericarp of the fruit and the seed coat are fused (Esau, 1977). Although the thickness of the pericarp varies, in sorghum it shows relatively little deformation; hence, here, we use the terms ‘fruit shape’ and ‘seed shape’ interchangeably when referring to phenotype at the macroscopic level.

Domesticated sorghum exhibits kaleidoscopic variation in inflorescence forms and seed shapes generated by millennia of human selection and interchange of germplasm (Harlan & De Wet, 1971). Attempts at formal Linnaean classification led to a bewildering taxonomy of species, subspecies, varieties, and forms (Snowden, 1936, 1955) that obscured the interfertility and cytogenetic similarity of the various taxa (De Wet, 1978). To generate a more practical classification, Harlan & De Wet (1972) included nearly all of Snowden’s taxa in a single, highly polymorphic species, *S. bicolor*, which was then divided into five morphological groupings referred to as races – Bicolor, Caudatum, Durra, Guinea, and Kaffir – plus an additional 10 intermediates. To distinguish among the races, they then developed a scoring system based heavily on seed shape and inflorescence morphology (Supporting Information Fig. S1a,b). The Harlan and De Wet classification receives some support from DNA sequence data (Morris *et al.*, 2013; Zhang *et al.*, 2015), but it is also clear that the races overall are not discrete entities, with Bicolor in particular being especially heterogeneous. A few of the races correspond to geographic regions; for example, Guinea is most commonly found in western Africa, and Durra‐like plants occur in eastern Africa and western India (Deu *et al.*, 1994; Morris *et al.*, 2013). Although several studies have investigated inflorescence architecture in sorghum (Brown *et al.*, 2006; Zhou *et al.*, 2019), these have not attempted to evaluate the characteristics suggested by Harlan & De Wet (1972), in part perhaps due to the difficulty in measuring such features (Fig. S1). Furthermore, the morphological diversity among the races has not been investigated rigorously using contemporary quantitative methods, leaving this an open area for investigation. In particular, we considered whether high‐dimensional phenotyping and multivariate analysis would mimic the relationships seen based on genetic background, or if greater morphological diversity or alternative patterns would be observed instead.

## Materials and Methods

### Plant materials and growing conditions

Germplasm representing the Sorghum Association Panel (Casa *et al.*, 2008) was planted in single‐row plots in June 2017 at the University of Missouri Columbia Bradford Research Center (latitude 38.89°N, longitude −92.20°W). Within each plot from a single field replicate, a randomly selected panicle was harvested in October 2017 to be used in analysis. For this study, only accessions whose botanical race assignment was clearly supported by genetic evidence were considered (Casa *et al.*, 2008). Principal component analysis (PCA) of the genotypic data presented by Morris *et al. *(2013) was used to further confirm accessions that corresponded best to one botanical race. We chose 55 representative accessions, comprising 11 Bicolor accessions, 15 Caudatum, 10 Durra, 9 Guinea and 10 Kafir (Table S1). We harvested an additional panicle from a second field replicate for 34 accessions. Harvested panicles were placed upright in large boxes to prevent flattening or overall distortion of panicle shape.

### Imaging by X‐ray computed tomography

**Fig. 1:**
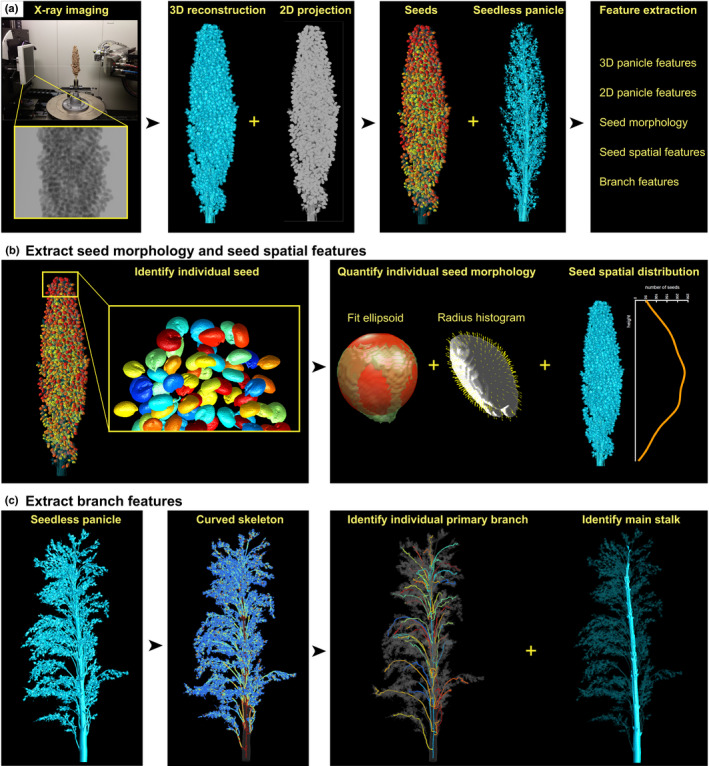
Pipeline to computationally dissect and quantify *Sorghum bicolor* panicles from three‐dimensional (3D) X‐ray imaging. (a) From X‐ray imaging, a 3D reconstruction and a composite side‐view two‐dimensional (2D) projection of the panicle were generated. Each individual seed is then identified and digitally removed to form a seedless panicle. Five categories of features can be extracted: 3D panicle features, 2D panicle features, seed morphology, seed spatial features, and branch features. (b) Pipeline for extracting seed profile: based on the intensity of the 3D X‐ray imaging, each individual seed can be captured and quantified by fitting an ellipsoid and computing the histogram of the radius along uniformly and densely distributed directions. Seed spatial features, such as seed number distributed along the panicle, can be measured by a curve. (c) Skeleton‐based pipeline for obtaining branch features: thresholding the X‐ray imaging generates a shape that is thinned to its curve skeleton. Then, geometric algorithms are used to extract the main stalk and primary branches, and their corresponding measurements.

A helical scan using the North Star Imaging X5000 system and the included efx‐dr software (MathWorks, Natick, MA, USA) was performed on individual panicles (clamped vertically upright), with 1000 radiographs generated per sample rotation, for a total of 6000–8000 radiographs depending on the length of the panicle, and an average helical pitch of 78.94 mm. The X‐ray source was set to a voltage of 70 kV, current of 1000 µA, and focal spot length of 70 μm. The radiographs were then combined using the North Star Imaging efx‐ct software to generate a 3D reconstruction of each sample, with a final voxel size of 106–109 µm. Three‐dimensional reconstructions were then converted to slices for import into Matlab and downstream processing (Figs 1a, S2a).

### Image processing and feature extraction

To reduce computational time, the margin space and parts of the stem approximately 1 cm below the first (lowermost) node were cropped out. The grayscale intensity was then adjusted and standardized automatically with a marker (a single stem sample used across all scans) or semi‐automatically without the marker (Fig. S2a). After adjusting and standardizing the grayscale intensity, a fixed and learned threshold (a grayscale value that is generally larger than air values and smaller than panicle values) was applied to segment out the sorghum panicle for feature extraction. The 3D object consisted of voxels each having three coordinates. From this, the eight features PanicleVolume, PanicleDepth, PanicleMaxWidth, PanicleWidthDepthRatio, PanicleConvexHullVolume, PanicleSolidity, PanicleElongation, and PanicleFlatness were calculated to measure the global shape of the 3D panicle. From the 3D reconstruction, a composite side‐view 2D projected image was generated. From this, nine 2D panicle features were calculated: Panicle2DArea, Panicle2DMajorAxisLength, Panicle2DMinorAxisLength, Panicle2DAspectRatio, Panicle2DConvexHullArea, Panicle2DSolidity, Panicle2DPerimeter, Panicle2DDepth, and Panicle2DCircularity. This 2D projection contains similar information to that in conventional 2D photograph imaging, except color (Table S2).

To extract seed morphological features, a fixed and learned upper threshold value was used to segment the seeds. Any small piece less than one‐eighth of the mean seed volume was treated as noise and removed. The shape of each seed was also used to remove outliers, such as very long pieces. When seeds touched each other, an erosion and dilation step in which seeds were shrunk and re‐expanded (also known as morphological opening; Fig. S2b) was applied to identify each individual seed. Seed morphological features included SeedNumber, SeedNumber/Depth, SeedTotalVolume, and SeedAvgVolume. Other seed morphology features were computed from 100 sampled seeds (Table S2). For this, each panicle was divided into 10 bins along the rachis, and within each bin 10 seeds were randomly picked from those whose volume was larger than the 25^th^ percentile (to avoid measuring underdeveloped seeds). SeedElongation and SeedFlatness were measured in two different ways: (1) computing the ratio of the voxel variation, and (2) fitting an ellipsoid. We also treated the distance between the ellipsoid and seed (SeedEllipsoidError) as a seed morphology feature (Table S2). Since seeds are nearly ball‐like shapes, we computed the histogram (SeedshapeRhist1‐10) of the radius along uniformly and densely distributed directions (Figs 1b, S3). We identified and recorded the centroid position of each seed, allowing us to calculate the 3D spatial distribution. The distributions of seed number, total seed volume, and average seed volume along the panicle are discretized as SeedNumbervhist1‐10, SeedBiomassvhist1‐10, and SeedSizevhist1‐10 (Figs 1b, S3). The distances between seeds and the main stalk permitted computation of three derived traits: MinDistanceSeedMainStalk, AvgDistanceSeedMainStalk and MaxDistanceSeedMainStalk.

Rachis, primary branch, and node features were extracted through a computational pipeline involving the medial axis. Each seedless panicle volume was downsampled by 2, and then an intensity threshold was applied in order to eliminate noise and generate a 3D shape (Fig. S4). A hole‐filling algorithm was applied to the shape in order to consolidate the stem into one single skeleton curve when the medial axis is computed. The trait MainStalkDiameter is computed as a by‐product during this step. This shape was thinned to its 2D medial axis using the Voxelcore method (Yan *et al.*, 2018) and further down to a one‐dimensional (1D), noise‐pruned, curve skeleton using the erosion thickness method (Yan *et al.*, 2016). The resulting skeleton is also equipped with a radius measure. The stem is identified by applying hysteresis thresholding of the radius measure to the skeleton to obtain a thick region, and then finding a high radius path through this region (Fig. S5). The junctions along the stem form nodes, from which a search algorithm is applied to identify candidate primary branches (Fig. S6). From the stem, node, and branch identification steps are derived seven traits: 1°BranchNumber, 1°BranchNumber/Depth, First1°BranchLength, 1°BranchAvgLength, LongestInternodeLength, 2ndLongestInternodeLength, and 1°BranchTipAngle (Table S2). Internodes were defined as the distance between two adjacent primary branch nodes, regardless of whether these internode sections are elongated (e.g. panicles with more evenly distributed nodes, or panicles with clustered nodes resembling whorls).

Additional information and detailed feature calculations are described in Methods S1. Raw extracted measurements from every sample are available in Table S3.

### Statistical analysis

For statistical analysis, phenotypic values of replicates were first averaged for each accession (unless otherwise indicated). For differences in means in univariate traits between botanical races, a Kruskal–Wallis test was first performed; traits with significant differences between any of the botanical races were then tested between every pairwise combination of races using a Mann–Whitney *U* test. Similarly, for homogeneity of variances between botanical races in univariate traits, a Bartlett test was first performed; traits with significant differences in variance between any of the botanical races were then tested between every pairwise combination of races with the Brown–Forsythe test using the *leveneTest* function with center = ‘median’ in the R package car (Fox & Weisberg, 2019). For each statistical test, *P*‐values across all phenotypes were adjusted together using the Benjamini–Hochberg method. For distribution (binned) traits, each distribution was treated as a 10‐dimensional vector. To determine statistical significance between the distributions of any two races, 1000 random permutations were run. At each permutation, the mean vector was calculated for each race, and the distance (*X_i_*, where *i* is a single permutation) between mean vectors of two races was calculated by using the *L*_2_ metric. The *P*‐value was calculated as the proportion of sampled permutations with *X_i_ *> *X*_0_, where *X*_0_ is the original difference in means between the two races.

PCA and hierarchical cluster analysis were performed in Matlab using functions *pca* and *clustergram*. The R function *cor.mtest* and package corrplot (Wei & Simko, 2017) were used for Spearman correlation significance tests and correlation matrix visualization. The function *lda* in R package mass (Venables & Ripley, 2002) was used for linear discriminant analysis (LDA) with a jackknifed ‘leave‐one‐out’ cross‐validation (LOOCV) method on a per‐accession basis to calculate classification accuracy. The function *mantel* in R package vegan (Oksanen *et al.*, 2018) was used to perform Mantel tests, which compares the correlation between two matrices. This test was used to detect possible genotype–phenotype associations, with genotype being represented by the kinship matrix from Shakoor *et al. *(2016).

For the other classification methods, parameter tuning and LOOCV were performed using the R package caret (Kuhn *et al.*, 2019). For a support vector machine (SVM), the classification function within the R package kernlab (Karatzoglou *et al.*, 2004) was used with method = ‘svmRadial’ (which outperformed ‘svmLinear’ or ‘svmPoly’) and a parameter search including sigma = seq(0.001, 0.1, by = 0.001) and C = 1:30. For random forest, the classification function within the R package randomforest (Liaw & Wiener, 2002) was used with a parameter search including mtry = 1:20 and ntree = 1000. For naive Bayes, the classification function within the R package naivebayes (Majka, 2019) was used with a parameter search including laplace = c(0,1) and usekernel = c(T,F). For *k*‐nearest neighbors, the classification function within the R package kknn (Schliep *et al.*, 2016) was used with a parameter search including kmax = 1:30, distance = 1:10, and kernel = ‘optimal’.

The *k*‐means clustering for two, three, four, or five clusters was performed using the base R *kmeans* function, with the parameter nstart = 25. Average cluster silhouette values and visualization of *k*‐means clustering were generated using the *fviz_nbclust* and *fviz_cluster* functions, respectively, from the R package factoextra (Kassambara & Mundt, 2017).

### Workstation, computational time, and code availability

We ran our pipeline on two different workstations. Depending on the dimension of the image volumes, it took between *c.* 20 min and several hours to calculate all the digitally measured traits, excluding branch traits, on an x64‐based PC with an Intel Xeon E5‐2630 CPU at 2.20 GHz. For primary branch and rachis detection and trait extraction, the running time ranged between 2 and 5 min on an x64‐based PC with an Intel Xeon W3690 CPU at 3.47 GHz.

The full 3D imaging dataset for this work can be downloaded from: https://www.danforthcenter.org/scientists‐research/principal‐investigators/chris‐topp/resources. All code used for image processing, digital feature extraction, and statistical analysis from this study can be found at the following GitHub repository: https://github.com/Topp‐Roots‐Lab/3D‐Sorghum‐Inflorescence.

## Results

### Three‐dimensional imaging and digital phenotyping of sorghum inflorescences using X‐ray tomography

To quantify inflorescence traits and their variation among the five main botanical races of sorghum, we imaged individual panicles using XRT (Fig. S1). The average scan time was approximately 10 min per sample, making this approach amenable to moderate throughput. From the Sorghum Association Panel (Casa *et al.*, 2008), 34 accessions were selected for imaging with two field replicates each. Subsequently, an additional 21 accessions were imaged with a single replicate each, for a total of 55 sorghum accessions included in this study, consisting of nine or more accessions from each botanical race. Accessions were chosen solely based on high genetic support for identity within their respective botanical races (Table S1), not by any phenotypic criteria, thereby selecting genotypes that should be unambiguously among the best genetic representations of each botanical race. Three‐dimensional panicle features, including global shape and size, were then computed in Matlab using the image volumes generated from an automated reconstruction algorithm after adjusting the grayscale intensity (Fig. S2a). For comparison, a composite side‐view 2D projected image was generated from the 3D reconstruction to measure 2D shape and size, features that more closely resemble traits typically measured in conventional photographic imaging of sorghum panicles (Fig. 1a).

A major advantage of using XRT for inflorescence phenotyping is the simultaneous capture of seeds and their 3D shape profile. To identify individual seeds, a threshold (a grayscale value that is generally larger than branch values and smaller than seed values) was first used to segment the seeds relative to branch segmentation. When seeds touched each other, seeds were computationally shrunk and re‐expanded to identify a morphological gap separating individual seeds. Seed morphological features were calculated by various approaches, including fitting an ellipsoid to the 3D image (see details in the Materials and Methods section). Since seeds are roughly spherical, we computed the histogram of the radius along uniformly and densely distributed directions to extract finer details in seed shape than 2D images can capture (Figs 1b, S3a). Because the positions of all the seeds were recorded, we were also able to compute seed spatial features, such as the distribution of seed number, total seed volume, average seed volume along the panicle (Figs 1b, S3b), and the distances between seeds and the main stalk (rachis).

Next, we developed a skeleton‐based method based upon the medial axis (Blum, 1967) to measure features related to the rachis, primary branches, and nodes after digitally removing the seeds (seedless panicle; Figs 1c, S7). The thin, homotopy‐preserving properties of the skeleton allow our method to efficiently obtain morphological branch traits while preserving the topology of the digital panicle shape; the thickness measure along the skeleton was then used to identify the main stalk, along which nodes were identified. The nodes, which form junctions within the skeleton, were used as starting points for a search algorithm to identify primary branches. Branch features were then derived from the identified stem, nodes, and primary branches.

In total, our methods retrieved 77 inflorescence and seed features (37 stand‐alone and 40 distributional), more than any other quantitative study on sorghum inflorescence phenotype to date; a full list of features, detailed descriptions, and sample measurements are shown in Methods S1 (Tables S2, S3).

### Phenotypic validation and relationships

**Fig. 2:**
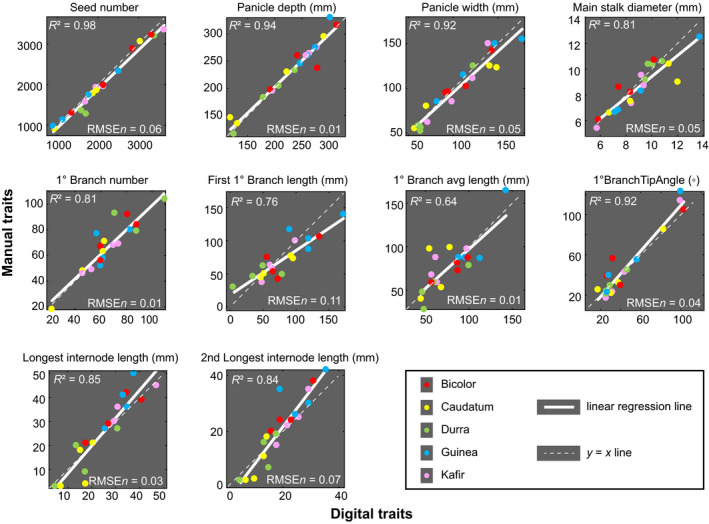
Comparison between digitally and manually measured traits in *Sorghum bicolor* panicles. Linear regression is performed for 10 features, with points representing individual panicles and colors representing the different botanical races. The white solid line is the regression line, with *R* ^2^ value and normalized root‐mean‐square error (RMSEn) shown. The white dashed line indicates theoretical perfect correspondence.

To compare and validate the traits that could be estimated manually, we measured 10 traits by hand for 20 individual panicle samples (*c.* 1.5 h per sample, Methods S1) and calculated the coefficient of determination *R* ^2^ with their digital counterparts (Fig. 2). Three traits commonly and easily measured in breeding and genetic studies – seed number, panicle depth, and panicle width – were highly consistent between manual and digital methods, with *R* ^2^ values of 0.98, 0.94, and 0.92, respectively. Main stalk (rachis) diameter had a weaker correspondence between manual and digital methods (*R* ^2^ = 0.81), likely due to the rachis cross‐section often being more ellipsoidal or irregular rather than a perfect circle, creating uncertainty in the manual measurements. The remaining manually measured features related to primary branch or internode traits and had *R* ^2^ values between 0.64 and 0.92. Here again, traits that could more easily be measured by hand (e.g. primary branch tip angle) showed stronger correspondence between manual and digital methods (*R* ^2^ = 0.92), whereas more tedious or difficult measurements such as longest internode length or second longest internode length (where exhaustive manual searches over the entire rachis are infeasible) showed lower correspondence, with *R* ^2^ values of 0.85, and 0.84 respectively. Indeed, most of the digital measurements are likely more accurate than manual measurements, except for primary branch number and first primary branch length, where the digital measurement has relatively higher uncertainty due to challenges in identifying and segmenting all primary branches. Similarly, average primary branch length showed a lower correspondence (*R* ^2^ = 0.64) possibly due to difficulties in both digital and manual measurements, and manual measurements took the average of nine random branches as opposed to the larger proportion of branches measured by the digital method. Overall, however, the consistency between digital traits and manually measured traits is high enough to give confidence in the measurements, with most instances of lower correspondence being explainable by the aforementioned challenges, primarily among manual methods.

Next, we computed the relationship between each pair of the 77 digital traits using Spearman’s rank correlation coefficient *ρ*, with each accession being an observation. From this, we confirmed several intuitive or expected correlations, such as between 3D panicle width and 3D panicle convex hull volume (*ρ* = 0.95, *P* = 1.18 × 10^−28^), and between panicle flatness and panicle width (*ρ* = −0.49, *P* = 1.32 × 10^−4^), which implies flat, wide panicles are more common than round, broad ones (Fig. S8). Total seed number was positively correlated with total seed volume (*ρ* = 0.75, *P* = 3.16 × 10^−11^), but slightly negatively correlated to average seed volume (*ρ* = −0.31, *P* = 0.02), indicating a generally inverse relationship between seed number and seed size in sorghum. On average, seeds also tended to be farther from the rachis when the panicle is longer (*ρ* = 0.71, *P* = 1.02 × 10^−9^). We also found relationships between traits that, though not surprising, are difficult to measure manually, such as between the longest internode length and average seed‐to‐rachis distance (*ρ* = 0.47, *P* = 3.35 × 10^−4^), and the inverse correlation between 3D panicle solidity and 3D panicle convex hull volume (*ρ* = −0.83, *P* = 9.26 × 10^−15^). Finally, our measurements also indicate that seed shape is generally independent of other panicle features.

Giving confidence to our digital measurements, correlations between 3D features and the closest analogous 2D features were high, such as in the case of 3D convex hull volume and 2D convex area (*ρ* = 0.95, *P* = 5.45 × 10^−29^), or 3D panicle solidity and 2D panicle solidity (*ρ* = 0.95, *P* = 1.78 × 10^−29^, Fig. S8). A representative variety of topological structures is shown in Fig. S7. The primary branches and rachis, shown in different colors, are computed using our skeleton‐based method. The negative correlation between primary branch density and panicle depth (*ρ* = −0.62, *P* = 3.56 × 10^−7^) is verified by the common observation of panicles that were either short and dense or long and sparse. Denser primary branches correlated with shorter internode lengths (*ρ* = −0.70, *P* = 2 × 10^−9^, between primary branch density and longest internode length), whereas wider panicles tend to have greater tip angles and lower primary branch densities along the stem. This suggests that open branches would result in wider and sparser panicles, and that panicles with greater tip angles and branch density also have lower internode lengths. Note that our primary branch detection algorithm performs better for wider panicles that have fewer intersections between branches.

### Archetypal panicle morphology for major sorghum botanical races

**Fig. 3:**
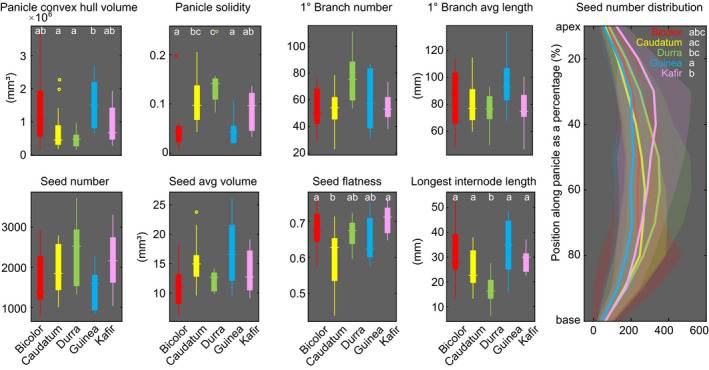
Distribution and variation for selected traits among the five botanical races in *Sorghum bicolor*. For boxplots, each dot indicates an outlier beyond the first/third quartiles ± 1.5 times the interquartile range. The central horizontal line indicates the median, the bottom and top edges of the box represent the 25^th^ and 75^th^ percentiles, respectively, and the whiskers extend to the most extreme nonoutlier data point. Each curve in the rightmost column represents the average seed number distributed along the panicle from apex to the base for each race. The shadow shows the region within one SD. Colors correspond to races; different letters indicate groups with statistically significant differences at *P* < 0.05.

Each of the five major botanical races is commonly associated with a stereotypical inflorescence phenotype, sometimes related to its most prevalent growing conditions (Harlan & De Wet, 1972). For example, Guinea panicles are frequently ‘open’ in overall architecture and thus presumably more resistant to mold or pests in wet conditions, whereas Caudatum and Durra panicles are frequently compact due to drier conditions and therefore potentially more amenable to selection purely for high yield. To provide a comprehensive empirical evaluation of these associations, we aggregated the genotypes by genetically defined botanical race and compared trait averages and variances (Figs 3, S9) for the 37 stand‐alone traits (i.e. not a distribution across the panicle or seed sections). For 14 of these, at least one of the five botanical races was significantly different from the others (Kruskal–Wallis test and Mann–Whitney *U* test, *P* < 0.05; Table S4). For example, within 3D panicle traits, convex hull volume differed significantly between Guinea and Caudatum or Durra; solidity differed significantly between Durra and Bicolor, Guinea, or Kafir, and between Caudatum and Bicolor or Guinea (Fig. 3). Branch traits also differed between botanical races; for example, Durra was significantly different from the other four races in primary branch density, first primary branch length, and longest internode length. In seed traits, notable differences were identified, including differences in seed number density (Caudatum vs Durra or Guinea, Durra vs Bicolor or Guinea, and Guinea vs Kafir), seed flatness (Caudatum vs Bicolor, Durra, or Kafir), and seed ellipsoid error, which is the distance between the seed and its fitted ellipsoid (Caudatum vs Bicolor, Guinea or Kafir, and Durra vs Bicolor, Guinea, or Kafir). Overall, Durra vs Guinea differed in the greatest number of stand‐alone traits (12), followed by Durra vs Bicolor (10), then Durra vs Kafir or Caudatum vs Guinea (both eight). Seven traits also differed in variance between at least one pair among the botanical races (Bartlett test, *P* < 0.05), and within these traits all but one had significant differences in variance between two or more pairs of botanical races (Brown–Forsythe test, *P* < 0.05; Table S4). Together, this indicates that our 3D phenotyping pipeline is sensitive enough to detect fine‐scale differences between the various types of sorghum.

Next, we treated each of the four sets of distributional traits (10 features each) as a vector and tested for significant differences between botanical races using 1000 random permutations (Figs 3, S9). For seed number distribution, Kafir is significantly different from Caudatum or Guinea, and Caudatum and Guinea are different from each other (Table S5). For seed biomass distribution, Bicolor is significantly different from all the others but Guinea; and Caudatum is different from Kafir. For seed size distribution, Guinea is significantly different from all the others but Caudatum; Caudatum is different from Bicolor or Durra. The most striking result is that all pairs of races, except Durra vs Guinea, differ statistically for the seed shape radius histogram, implying that seed shape is one of the main morphological features to distinguish between races. Furthermore, it demonstrates the importance of employing new 3D imaging techniques and advanced morphometrics for phenotyping, since 3D seed shape is challenging and time‐consuming to quantify by any manual method.

**Fig. 4:**
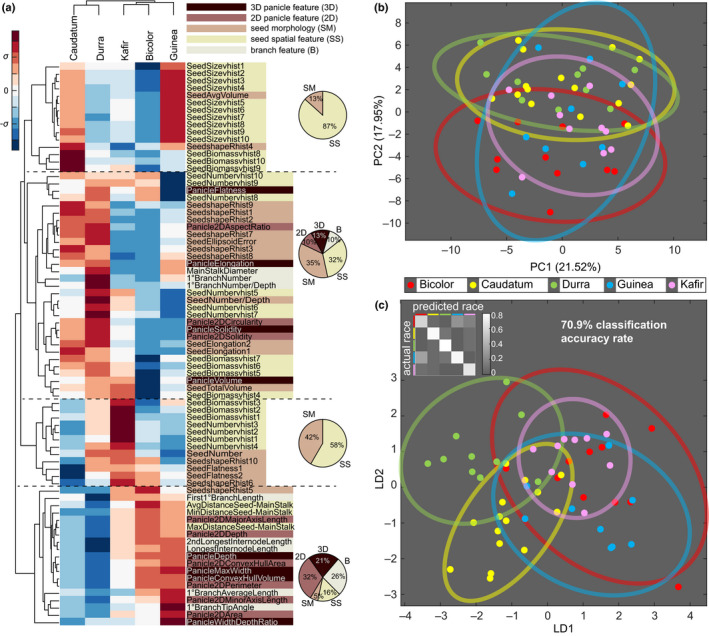
Two‐way hierarchical cluster analysis, principal component analysis (PCA), and linear discriminant analysis (LDA). (a) Two‐way hierarchical cluster analysis based on the mean value of morphological features (rows) among the five botanical races of *Sorghum bicolor* (columns). The heat map indicates an increase (red) or a decrease (blue) with respect to the overall mean value of five races. The features are clustered into four main groups. The pie charts show the composition of feature categories for each group. (b) PCA plot of all morphological features with 85% confidence ellipses. The percentage variance for each principal component (PC) is shown in parentheses. Colors indicate botanical race. (c) LDA plot on the first eight PCs (84.3% variance) with 85% confidence ellipses. Colors the same as in (b). The confusion matrix for predicted species is shown in the upper left corner. Jackknifed ‘leave‐one‐out’ cross‐validation found 70.9% classification accuracy.

To compare the overall differences in inflorescence traits, we calculated the mean value of every trait according to botanical race and performed two‐way hierarchical clustering (Fig. 4a). Cluster analysis identified two major morphological groups made up of Caudatum plus Durra, and Kafir plus Bicolor and Guinea. Within the latter group, Kafir and Bicolor were more similar to each other than either was to Guinea. Four general clusters of traits could be identified. Two were comprised entirely of seed traits: one cluster (topmost 15 traits in Fig. 4a) was predominantly seed spatial features related to size, whereas the other cluster (12 traits, third cluster down in Fig. 4a) was more equally composed of seed spatial traits and seed morphology traits, including flatness, shape, and number. However, when the botanical races are compared, the relative values of traits of the two seed‐dominated clusters were nearly opposite of each other: Caudatum and Guinea had higher values for the first cluster, including seed size distribution and seed biomass distribution in the lower half of the panicle, whereas Durra and Kafir had higher values for the second cluster with seed number, shape distribution, and seed biomass distribution in the half top panicle. The other two clusters of traits were composed of a mix of panicle features, branch features, and seed traits, although seed traits still made up more than half the total for one of these two. This suggests that seed traits can differentiate between genetically defined botanical races.

### Panicle variation within botanical races prevents robust pedigree‐based classification

To assess variation both within and between botanical races across all phenotypes, we performed PCA using all 77 traits (Fig. 4b). The first principal component was roughly split across all types of traits (2D/3D panicle features, seed morphology and spatial features, and branch features), indicating that all trait types contribute to the axis of greatest variation (Fig. S10). The specific traits include panicle 3D depth, volume, and solidity; 2D area, depth, and solidity; seed number, total volume, distance between seed to the rachis, and number/biomass distribution; and first and second longest internode length and primary branch number/depth. The second principal component, by contrast, was primarily based on seed morphology and spatial features, and particularly for individual distribution traits, such as seed shape radius histogram in bin 2 to bin 5 (Fig. S3). Notably, however, PCA did not generate obviously distinct clusters; although the centroids of clusters manually annotated by botanical race did show relative positions consistent with that of the hierarchical clustering, all clusters were partially overlapping with each other. We also did not observe any clusters using nonlinear dimensionality reduction techniques such as Isomap (Fig. S11). This lack of distinct clusters using an unsupervised approach suggests that the botanical races have no obvious phenotypic boundaries between one another based on overall morphological trait variation.

We next used supervised approaches: PCA–LDA, SVM, random forest, naive Bayes, and *k*‐nearest neighbor. PCA–LDA, using the values of the first eight principal components from all 77 traits (capturing 84.3% of the total variance) as inputs into an LDA, performed best (Table S6). Leave‐one‐out cross‐validation resulted in a 70.9% overall classification accuracy, which, though higher than expected by random chance, was still relatively modest given our strict genetically based selection process (Table 1). Unlike with PCA, the LDA loadings were predominantly influenced by seed morphology traits (Fig. S10), especially seed flatness and elongation, and clusters labeled by botanical race were more distinct, although still clearly intermixed. PCA–LDA was then performed based on the traits in each feature category (3D/2D panicle features, seed morphology/spatial features, branch features), with seed morphology traits performing best (Table 1), providing another line of evidence that seed morphology dominates the race classification. However, most panicles between the various botanical races were overlapping in feature space and not distinct from one another, as PCA–LDA was unable to fully separate the races. Additionally, *k*‐means clustering using up to five clusters showed no evidence of distinct clusters; even the overlapping groups failed to correspond to botanical race (Fig. S12).

**Table 1:** Results of principal component analysis–linear discriminant analysis (PCA–LDA) classification for the five botanical races in *Sorghum bicolor* using different categories of features, and Mantel tests for correlation between genotype and phenotype.

Trait category	No. of traits	Discriminant analysis		Mantel test with −kinship	
		8 PCs variance (%)	Accuracy rate (%)	Correlation *r*	*P*‐value
All features	77	84.3	70.91	0.1364	0.002
3D panicle features	8	100	29.09	−0.005127	0.503
2D panicle features	9	99.94	34.55	0.02546	0.227
Seed morphology	19	97.16	54.55	0.2333	0.001
Seed spatial features	33	95.14	45.45	0.0966	0.013
Branch features	8	100	36.36	0.03337	0.203

**Fig. 5:**
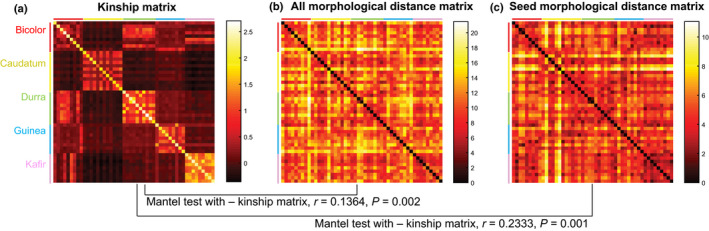
Mantel test comparing genotype and phenotype, with respect to *Sorghum bicolor* race. (a) The kinship coefficient matrix, created based on marker data (identity by state). (b) The pairwise distance matrix, based on all morphological features. (c) The pairwise distance matrix, based on seed morphological features. Mantel tests compared the (a) negative value of the kinship matrix against (b) and (c) distance matrices with *r* = 0.1364 and *P* = 0.002 and with *r* = 0.2333 and *P* = 0.001, respectively.

We considered whether phenotype reflected genotype independently of botanical race by comparing a kinship matrix of all accessions against distance matrices either based on all 77 traits or just seed morphology traits (Fig. 5). A Mantel test comparing the kinship matrix and the total morphological matrix found a slight but statistically significant correlation (*r* = 0.1364, *P* = 0.002), whereas comparing the kinship matrix with just the seed morphology matrix had a somewhat stronger, but still relatively low, correlation (*r* = 0.2333, *P* = 0.001). To determine whether this can be affected by feature selection, we performed similar comparisons using increasingly stringent subsets of traits for constructing the morphological matrix, based on the coefficient of determination *R* ^2^ between accession replicates (Table S7). Phenotypic differences among accession replicates may be due to innate biological (i.e. developmental) variation, as well as environmental variation present within the field sampling. However, the kinship‐to‐morphology Mantel test was not significantly changed either from removing the features that had lower between‐replicate consistency or by repeating the comparison using only replicate 1 or replicate 2 phenotypic data (Table S8). In conjunction with the results of PCA and LDA, this result indicates that morphological differences between either the botanical races or overall genetic background are quantitative rather than qualitative, and relatively modest. Instead, panicle morphology is more accurately described as a continuum of trait variation without obvious categorical distinctions.

## Discussion

Inflorescence architecture in grasses directly controls the number of spikelets, and therefore the number of seeds. In addition to its economic and ecological importance, inflorescence form has long been used to distinguish grasses from each other, and is a major character for distinguishing genera (Barkworth *et al.*, 2003; Wu *et al.*, 2006, 2007; Barkworth *et al.*, 2007). Unsurprisingly, grass inflorescence architecture has been the subject of numerous studies and reviews, addressing both morphological diversity (Vegetti & Anton, 1995; Perreta *et al.*, 2009; Reinheimer *et al.*, 2009; Kellogg *et al.*, 2013; Pilatti *et al.*, 2019) and its genetic control (Bommert *et al.*, 2005; Malcomber *et al.*, 2006; Kellogg, 2007; Bartlett & Thompson, 2014; Kyozuka, 2014; Whipple, 2017; Koppolu & Schnurbusch, 2019). Diversity among grass inflorescences can be summarized by several parameters: the number of orders of branching, the number of branches at each order, whether the inflorescence axis terminates in a spikelet, and the number of spikelets per branch (Kellogg, 2000; Doust *et al.*, 2005). In addition to variation in numbers of orders of branching, grass inflorescences vary in the length of the internodes. Internode elongation occurs late in development and is thus separable from branching pattern. Therefore, relatively small changes in orders of branching or internode elongation, which we have captured with XRT, can lead to inflorescences that look substantially different from each other.

Despite the rich literature describing inflorescence architecture in rice and maize, sorghum has received much less attention, either developmentally or genetically, although important recent papers include those by Brown *et al. *(2006), Morris *et al. *(2013), Hmon *et al. *(2014), and Zhou *et al. *(2019). Sorghum has three orders of branching before the spikelet pairs, and the primary branches form in a spiral phyllotaxis. However, the number of branches at order of branching, and the extent of elongation of internodes, is quite variable and challenging to capture in a quantitative way. Instead, the architecture of sorghum panicles is typically described using intuitive but imprecise and qualitative terms (De Wet, 1978).

Using recent advances in 3D phenotyping, we analyzed a diverse range of sorghum panicles, chosen solely based on their genetic background with no prescreening based on phenotype to bias the outcome. Though differences in the average panicle morphology between botanical races were detectable, multivariate analysis shows that panicle architecture is indeed continuous, and relying on discrete class descriptors should be avoided in situations where specificity is important. This morphological continuum could reflect local genetic introgression rather than broader genomic identity corresponding to race, potentially due to the complex history of breeding and local adaptation in domesticated sorghum. Although 2D image analysis may have come to a similar conclusion for some overall shape features (such as panicle area), our analysis using XRT 3D imaging additionally demonstrates a phenotypic gradient even for specific inflorescence features (such as branch lengths) that cannot be reliably measured using 2D imaging due to the occlusion of branches, which prevents accurate representation of the panicle branching hierarchy.

When compared with manual branch phenotyping methods, our skeleton‐based pipeline calculates morphological traits from branches that more completely and accurately represent each panicle. Our computational method nondestructively captures branches evenly distributed throughout the panicle, as opposed to hand‐picked branches that may be biased. By using the thinned, 1D curve structure of the skeleton, the topology of the shape and the semi‐automatic annotation of the stem and primary branches are more amenable for characterization, while retaining information of value. An additional advantage of digital characterization is improved objectivity, as manual measurement of traits such as branch angle, selection of the nine primary branches for average lengths, and node distances can be subjective. Although digital methods also need individual users’ choice of parameters, the space of such choices is significantly smaller than those in manual measurements.

In panicles with large tip angles, the longest‐path approach of our skeleton‐based method performs exceptionally well because of the lack of intersections between different branches within the shape. However, for compact panicles, the path of a single branch may appear to intersect with several others in the image. This may cause the detected branch to wind between several actual branches. Although branch length, curvature, tortuosity, and tip angle constrain the predicted branch path to be approximately correct, small deviations will inevitably cause parts of multiple branches to be included in the same path. In future work, we plan to explore methods from combinatorial optimization to better address such topological issues, potentially by clustering ‘branch‐like’ parts of the skeletons together.

Another advantage of XRT imaging for inflorescence phenotyping is that all the seeds within a panicle can be segmented and isolated with their entire 3D shape, a task that would otherwise be laborious, if not impossible, by optical imaging without the aid of robotics. Our analysis found that seed shape and size are among the more distinctive traits of the sorghum botanical races, thus illustrating the potential for high‐resolution mapping of seed traits. Additionally, within the genotypes analyzed, we found some support for a trade‐off between seed number and seed size, a long‐standing concept in biology that is often difficult to confirm empirically due to confounding factors (Smith & Fretwell, 1974; Paul‐Victor & Turnbull, 2009). However, because XRT imaging relies on object density, information such as the color of the seeds or glumes is not captured. Although there is little evidence that seed color is associated with botanical race, it may be of interest in other applications, such as predicting seed tannin content. On the other hand, glume morphology and the presence or absence of awns may be useful for distinguishing between sorghum botanical races (Harlan & De Wet, 1972). As they present special challenges for segmentation, we did not include them for analysis here, although future efforts may be able to incorporate these features.

Aside from these limitations, sorghum inflorescence phenotyping via XRT represents a more powerful, objective, and quantitative way of measuring panicle features compared with previous approaches. From the combined methods yielding 77 total inflorescence traits, we find interesting relationships between numerous features, but ultimately no compelling evidence that panicle architecture reflects botanical race. Indeed, in sorghum, botanical race is not a formal taxonomic rank and has no standing in a Linnaean classification, despite some support for genetic separation. In any case, our results suggest that the complex history and interbreeding of sorghum germplasm has resulted in panicle architecture overall being mostly dissociated from botanical race. This is potentially advantageous for applications such as genome‐wide association studies, in which population structure would be predicted to have less of an impact on genetic mapping. Likewise, we found no support for alternative categorical schemes (such as the common classification of panicle shapes into ‘open’, ‘closed’, ‘intermediate’, etc.) across our samples, indicating that, despite their convenience, they do not accurately reflect the quantitative nature of inflorescence variation present in sorghum. Future studies can be scaled to diversity panels or other mapping populations with reasonable expectations for finding potentially novel quantitative trait loci controlling the myriad of sorghum inflorescence features we have described here.

## Author contributions

CNT, EAK and TJ designed the experiment. M‐RS performed sample collection and imaging. ML, DZ and M‐RS performed feature extraction and analysis. M‐RS, ML and DZ wrote the manuscript with contributions from CNT, EAK and TJ. ML and M‐RS contributed equally to this work.

## Supporting information


**Fig. S1** Examples of sorghum panicle morphology.
**Fig. S2** Additional details on X‐ray imaging workflow.
**Fig. S3** Diagrammatic description of seed distribution features.
**Fig. S4** Thresholding considerations with primary branches.
**Fig. S5** Stem identification process.
**Fig. S6** Branch identification process.
**Fig. S7** Primary branches and rachises of representative sorghum panicles.
**Fig. S8** Trait correlation plot.
**Fig. S9** Distribution and variation for all traits among the five races.
**Fig. S10** Principal component analysis and linear discriminant analysis loadings.
**Fig. S11** Isometric feature mapping with all traits.
**Fig. S12** K‐mean clustering.
**Methods S1** Additional methods on primary branch and rachis trait extraction, skeleton generation, stem identification, primary branch identification and measurements, and manual panicle measurements.

**Table S1** Germplasm and genotypes.
**Table S2** Trait descriptions.
**Table S3** Complete trait value data.
**Table S4** *P*‐values for univariate traits.
**Table S5** *P*‐values for distribution traits.
**Table S6** Classification results from supervised methods.
**Table S7** Coefficient of determination between replicates.
**Table S8** Kinship‐to‐morphology Mantel test.
Please note: Wiley Blackwell are not responsible for the content or functionality of any Supporting Information supplied by the authors. Any queries (other than missing material) should be directed to the *New Phytologist* Central Office.
