# Supplementary material for: Comprehensive 3D phenotyping reveals continuous morphological variation across genetically diverse sorghum inflorescences
Source: New Phytol. 2020 Apr 16;226(6):1873–85. doi: 10.1111/nph.16533 (PMC7317572; doi:10.1111/nph.16533)

New Phytologist Supporting Information

Article title: Comprehensive 3D Phenotyping reveals Continuous Morphological Variation across Genetically Diverse Sorghum Inflorescences

Authors: Mao Li, Mon-Ray Shao, Dan Zeng, Tao Ju, Elizabeth A. Kellogg, Christopher N. Topp
Article acceptance date: 23 February 2020

The following Supporting Information is available for this article:

Methods S1

Primary Branch and Rachis Trait Extraction

Traits are extracted from a panicle which had its seeds digitally removed and the holes in its main stalk filled. As the first step, we thresholded the 3D image volume twice to produce two shapes for each panicle (**Fig. S4**). The first shape is segmented from the rest of the image by applying a lower threshold to the scan (**Fig. S4a**). By capturing all significant protrusions from the panicle's stem, the first shape provides for a reasonably accurate branch count and internode distance estimation. A second shape (**Fig. S4b**) is generated from a higher threshold in order to minimize the number of perceived intersections between branches. Our branch tracing algorithm performs more accurately with fewer of such intersections, and hence the high-thresholded shape is more useful for measuring branch length and tip angle. We explain the extraction of the stem, the primary branches and their traits below.

Skeleton Generation

To characterize the branches and other topological traits, we use a 1D curvilinear representation of the panicle that we call a *skeleton*. We start by generating the medial axis (Blum 1967), which is a 2D non-manifold surface structure for a 3D shape. Due to the complexity of the panicle and the voxelized nature of the data, we use a recently developed medial axis extraction method that offers the best efficiency among all existing algorithms and is specially tailored to voxel shapes (Yan *et al.* 2018). The Voxelcore method approximates the medial axis of the 3D shape by finding the interior Voronoi diagram of the shape's boundary vertices. The subset of Voronoi vertices, edges, and faces whose dual Delaunay elements have non-empty intersections with the shape make up the voxel core. We then reduce the resulting medial axis further down to a 1D structure, while pruning away spurious components of the medial axis due to irregularity of the shape (a known deficiency of the medial axis), using the method of (Yan *et al.* 2016). This method uses a significance measure, called Erosion Thickness, to identify 1D subsets of the medial axis that represents significant tubular features (e.g., panicle's stem and branches). Erosion thickness is defined as the difference between

the burn time of a fire over the medial axis and the maximal ball radii, where the burn time measures the lateral distance to the shape boundary along the medial axis. It thus captures the amount that the shape is shrunk after eroding the medial axis from its end points. The resulting skeleton is also equipped with a radius measure that captures the shortest distance from a skeleton vertex to the shape boundary.

Stem Identification

Before searching for primary branches, we first identify the stem (**Fig. S5**) using the radius measure along the curve skeleton. The stem identification algorithm, which is designed to capture continuously thick portions of the panicle, is detailed next.

1. The skeleton vertex with the largest radius, which corresponds to the thickest part of the stem, is chosen as a seed point. Neighboring vertices are added to a set S iteratively (**Fig. S5b**) until a reached vertex falls below a chosen radius threshold l_t, which is chosen so that S is ensured to include all of the stem but not too many of the neighboring points.
2. Kruskal's algorithm (Kruskal 1956) is applied on S to obtain a minimum spanning tree S_{mst} which is free of cycles (**Fig. S5c**).
3. Lastly, we find the stem S_{stem} as the longest path in the tree S_{mst} (**Fig. S5d**). This can be done in a number of ways. We use the following approach. The end-points of S_{mst} are iteratively removed until only a single point or a single edge are left in the tree. During removal, the iteration that each point is removed is recorded at that point. Lastly, we grow back the path from that single point or edge by iteratively adding back a point on an end of the path whose recorded removal iteration is one less than the iteration at the current path end.

Primary Branch Identification and Traits Calculation

Since primary branches come directly out of the main stem, our search for primary branches begins from junctions along S_{stem}, the subset of the skeleton which represents the stem. Beginning from the bottom of the stem (the side closest to the highest-radius vertex), we initialize searches from each junction on the stem. Note that some primary branches do not start from junctions directly on the skeleton path representing stem, but rather from junctions within a region surrounding the stem (**Fig. S6a**). As a result, we include all junction points on the skeleton whose distance to the nearest point p on stem is less than the radius measure recorded at p.

For each junction point, we trace a primary branch using the following heuristic. We first find the subgraph of the skeleton within d_{max} vertices away from the junction. Here, d_{max} is set high enough to capture the entire length of the branch, but not so high as to cause a candidate branch to weave between the subgraphs coming from several junctions. In practice d_{max} was set to between 800 and 1200 vertices depending on the size of the panicle. For each subgraph, we take the top-10% longest paths that start from the junction (**Fig. S6b**). This is based upon our observation that primary branches are usually longer than secondary branches. Among the longest paths, the one with the smoothest curvature is chosen to be a candidate primary branch from the junction (**Fig. S6c**). Our

definition of curvature of each path aims at capturing the total bending over all junctions along a path. It is computed as follows. An interval (set to 20 vertices wide along the path in our implementation) is fixed around each junction of the path. A sliding window (set to 6 vertices wide in our implementation) is rolled along this interval. For each window, the coordinates of the last and first window vertices are subtracted to result in a directional vector that approximates the tangent direction (the first derivative). The difference between the tangent directions of consecutive windows is then computed, whose L2 norm is taken as an approximation to the curvature (the second derivative). These differences are averaged across all pairs of consecutive windows along the interval to provide an average curvature value for that junction. The curvatures for all junctions are in turn averaged to produce one curvature value for the whole path.

After a single path is chosen among the candidate paths from the subgraph, tortuosity and tip angle are used to determine if the path is a valid branch. Firstly, if a candidate path's tortuosity is above a chosen threshold (typically 2), then the path would be too winding to represent a true branch. Tortuosity is computed as the ratio between the path's distance along the skeleton and the Euclidean distance between the path's start and end points. Secondly, if the tip angle is above a chosen threshold (typically 150 degrees), then the path likely winds back to the stem. The tip angle is calculated between the vector pointing from the bottom of the stem to the stem's centroid and the vector pointing from the path's start point to its end point. Both types of paths are removed.

Finally, the first and second longest internode distances are calculated as the top two longest distances between junctions along the stem with at least one valid branch.

Manual Panicle Measurements

Panicle Depth: The length of the rachis from 1 cm below the first (lowest) primary branch, to the panicle tip (apex).
Panicle Width: The maximum width of the panicle.
Main Stalk Diameter: The diameter of the rachis at 1 cm below the first primary branch.
Primary Branch Number: The total number of all primary branches along the panicle.
First Primary Branch Length: The length of the first (lowest) primary branch.
Primary Branch Average Length: Mean length of primary branches (one from each whorl).
Primary Branch Angle: Mean angle between the rachis and primary branches (one from each whorl).
Longest Internode Length: The longest internode length along the rachis of the panicle.
Second Longest Internode Length: The second longest internode length along the rachis of the panicle.
Seed Number: Total number of seeds, counted after panicle threshing.

SI References

Blum H. 1967. A transformation for extracting new descriptors of form, *Models for the Perception of Speech and Visual Form* pp. 362–80. MIT Press.

Kruskal JB. 1956. On the shortest spanning subtree of a graph and the traveling salesman problem. Proc. Amer. Math. Soc**.** **7**, 48-50

Yan Y, Sykes K, Chambers E, Letscher D, Ju T. 2016. Erosion Thickness on Medial Axes of 3D Shapes. ACM Trans. Graph. **35**(4), 38:1-12

Yan Y, Letscher D, Ju T. 2018. Voxel cores: efficient, robust, and provably food approximation of 3D medial axes. ACM Trans. Graph. **37**(4), 44:1-13

Fig. S1: Examples of sorghum panicle morphology. Scoring system developed by Harlan and De Wet based on seed shape (a), and inflorescence morphology (b) e.g. open (2, 3, 4), compact (5, 6, 7), half-broomcorn (8), and broomcorn (9) panicle head types. Representative samples for each genetically-defined botanical race (Bicolor, Caudatum, Durra, Guinea, and Kafir) used in this study (c); images are 2D projections derived from X-ray scans.

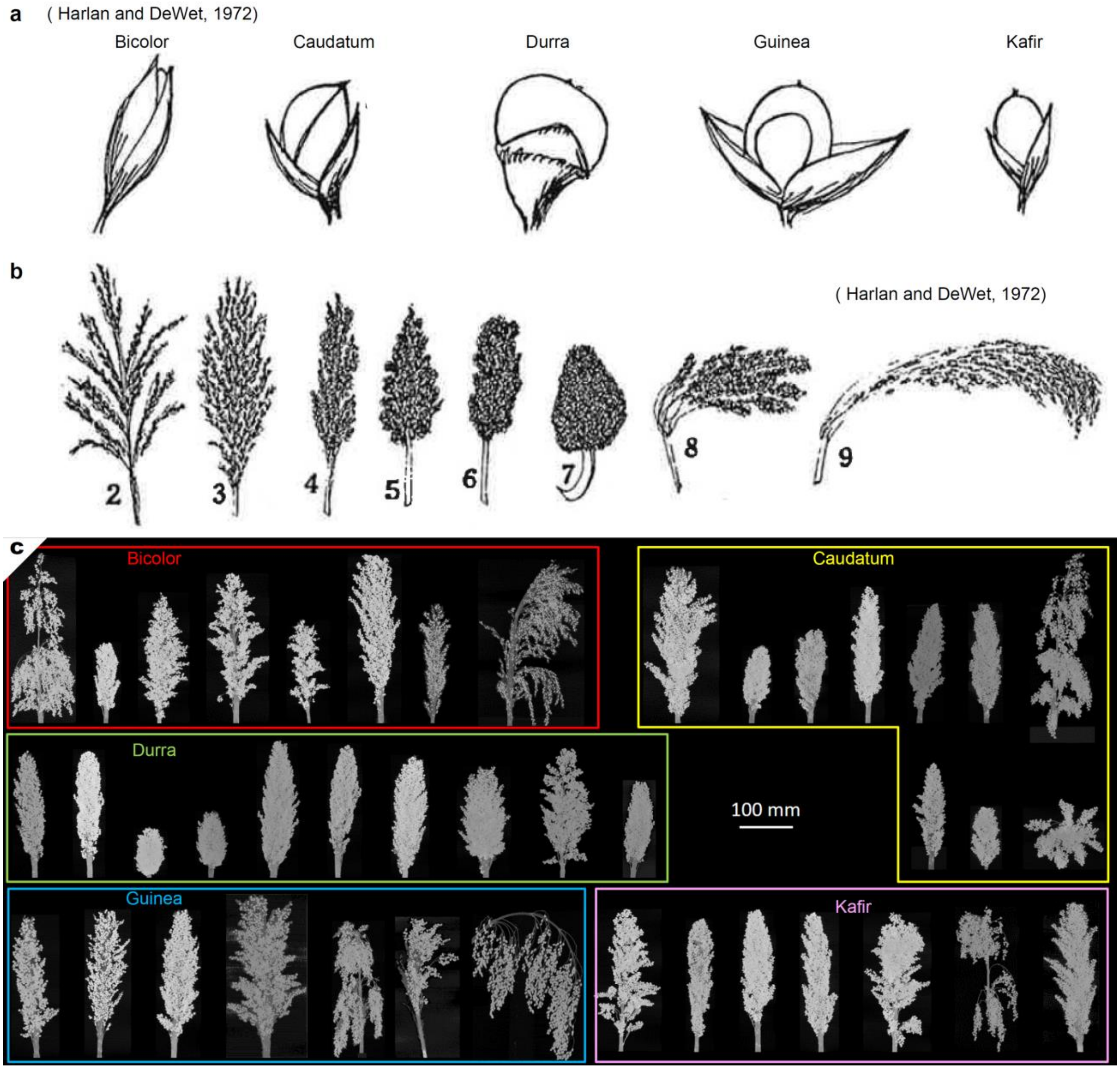

Harlan JR and De Wet JMJ. 1972. A simplified classification of cultivated sorghum. *Crop Sci.* **12**:172-176.

Fig. S2 Additional details on X-ray imaging workflow. (a) Illustration of pipeline including X-ray imaging; 3D reconstruction and export of slices; standardization based on a fixed length below the first node; adjustment of intensity based on a fixed density marker; and the resulting 2D and 3D images. (b) For seeds in physical contact (i.e. touching one other), an erosion and dilation step in which seeds were shrunk and re-expanded (also known as morphological opening) was applied to identify each individual seed.

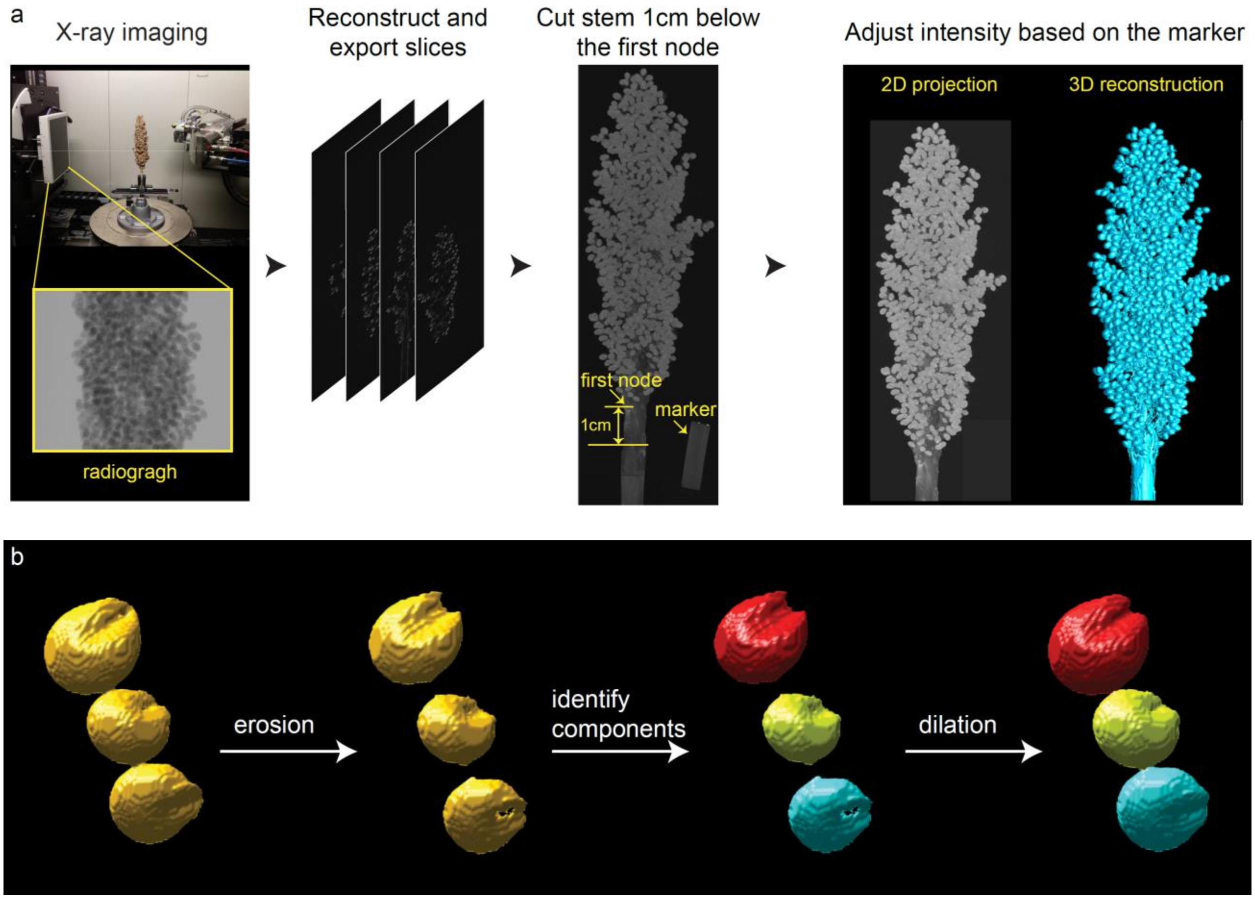

Fig. S3 Diagrammatic description of seed distribution features. (a) An example of 'seedshapeRhist' features in two contrasting samples. SeedshapeRhist is the histogram of radius (distance between surface and center) distributed along different directions. It was then discretized into 10 uniformly-sized bins ranging from smallest radius (blue) to largest radius (red). More flat seeds tend to have more spread histogram. (b) An example of seed number distribution along the panicle in two contrasting samples.

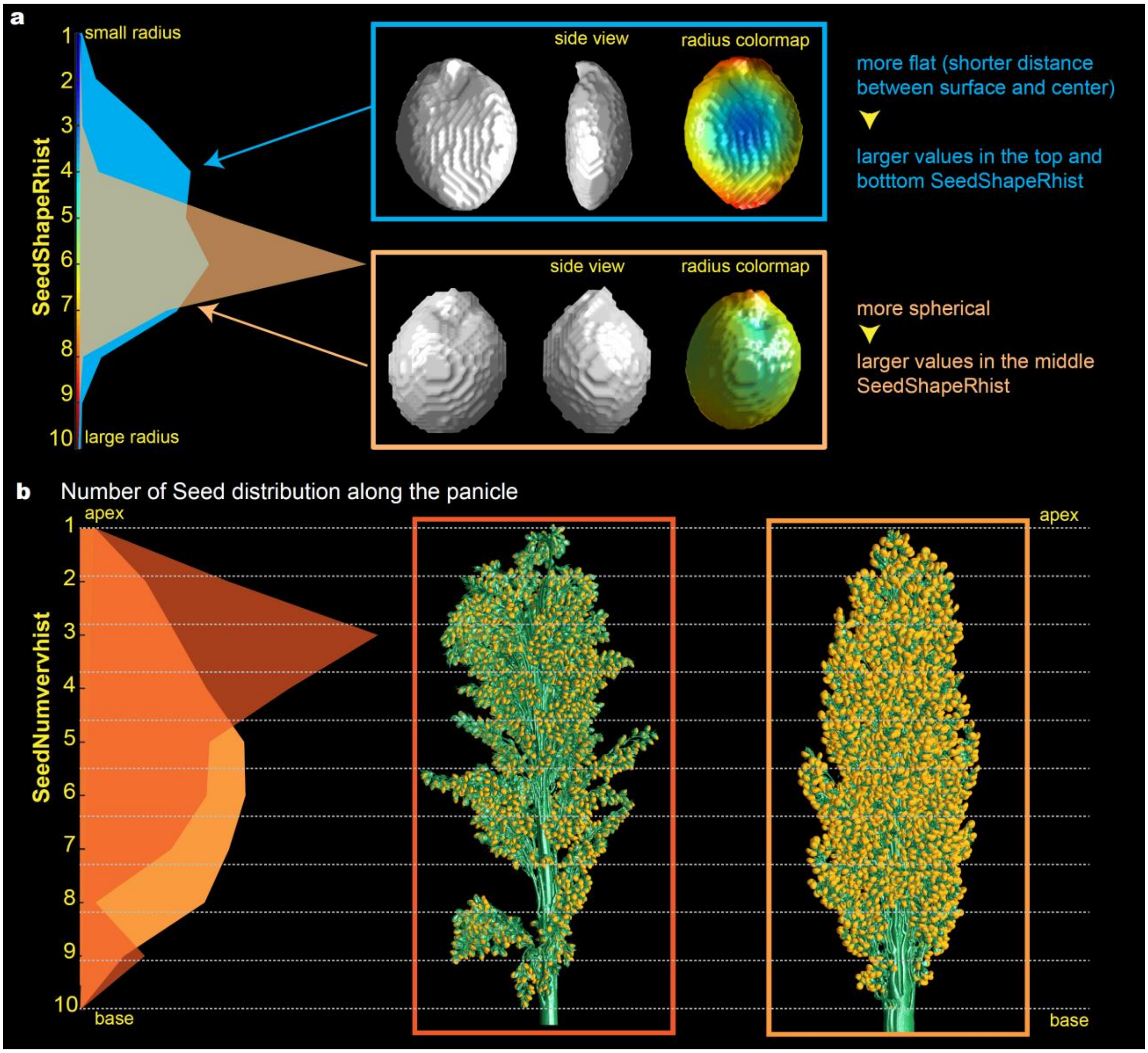

Fig. S4 Thresholding considerations with primary branches. At a lower threshold (a), all primary branches coming out of the stem are captured, but branches may merge after exiting the stem. Hence the lower thresholded shape is used to compute the branch count. At a higher threshold (b), gaps near the stem prevent all branches from being captured, but the fewer intersections allow our algorithm to more accurately trace the entire branch and measure branch length.

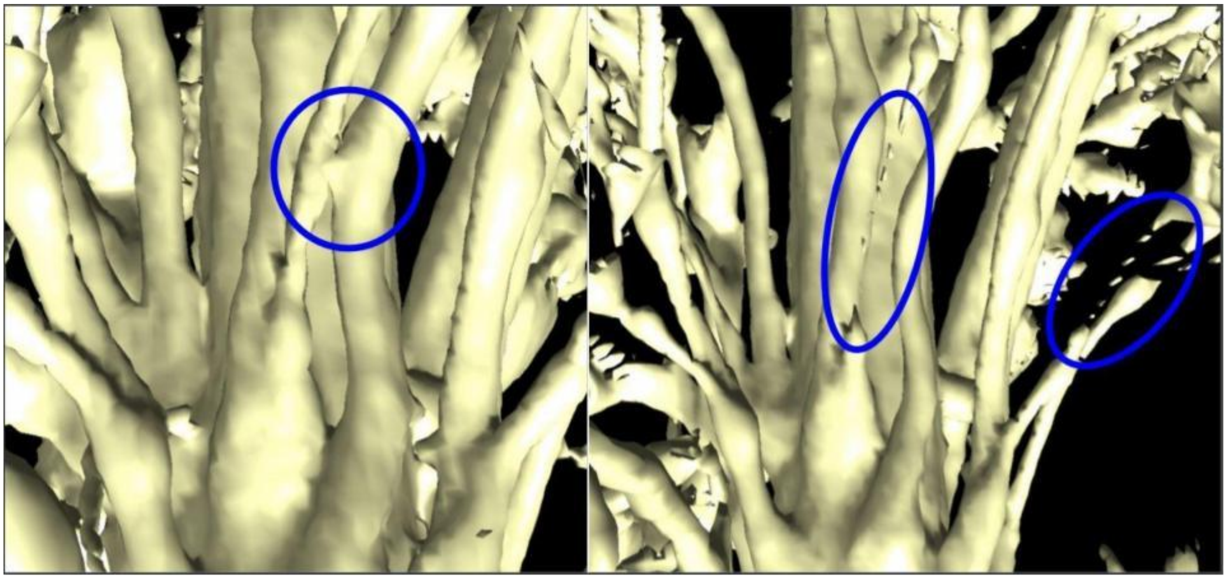

(a) (b)

Fig. S5 Stem identification process. (a) Panicle shape (gray) and the skeleton (colored by radius using the heat map, so that red means large radius). (b) A region of the skeleton is expanded from the source point with the highest radius (red circle near the bottom of the skeleton). (c) Cycles are broken using Kruskal's algorithm. Circled areas in close-ups show edges which are broken. (d) After finding the longest path, as described in the text.

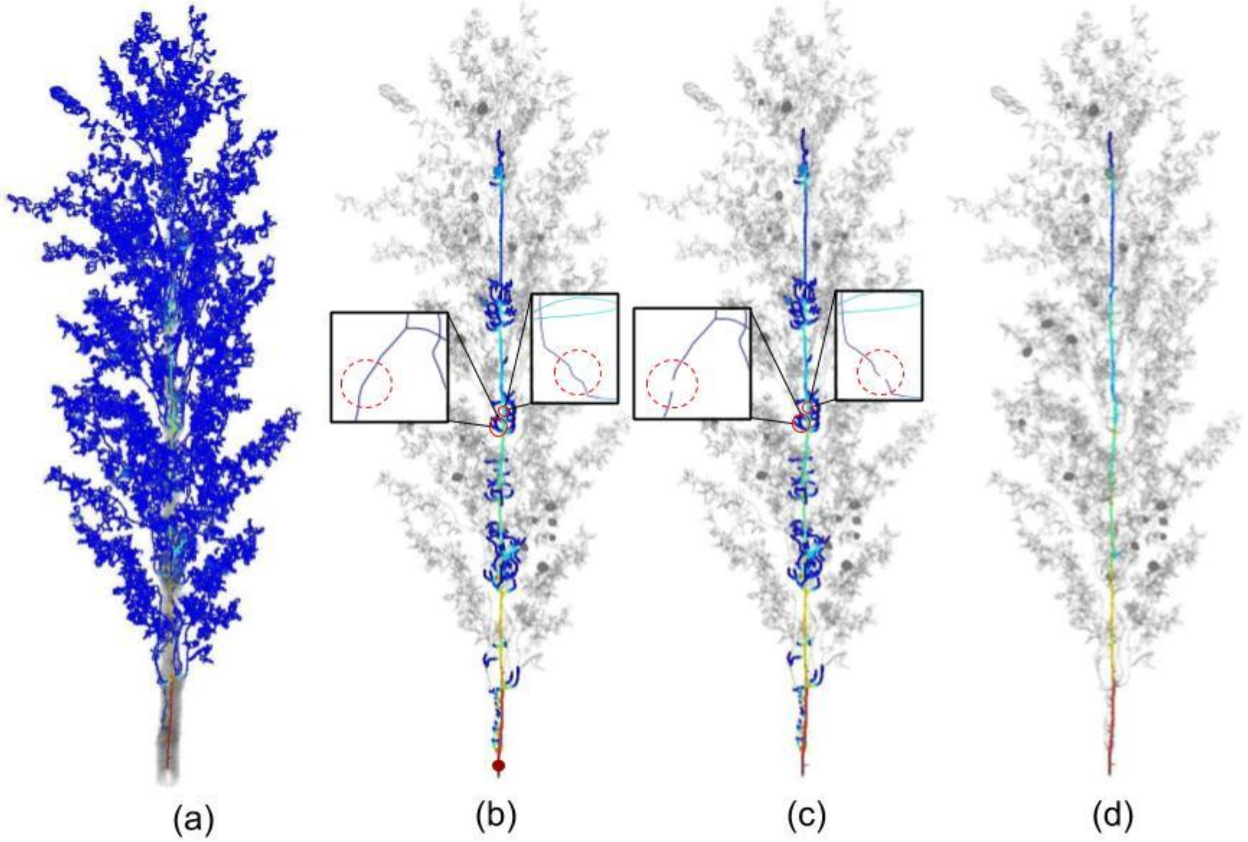

Fig. S6 Branch identification process. (a) Potential new branches begin both at junctions (red dots) along the stem's representation along the skeleton (S_{stem}, in black) and in nearby junctions within the stem. (b) Candidate branches are chosen from the top-10% longest paths (boxed) coming from each junction. (c) The path with the smoothest curvature that satisfies both the tortuosity and tip angle constraints is picked. All skeletons (except the stem) are colored by the radius measure.

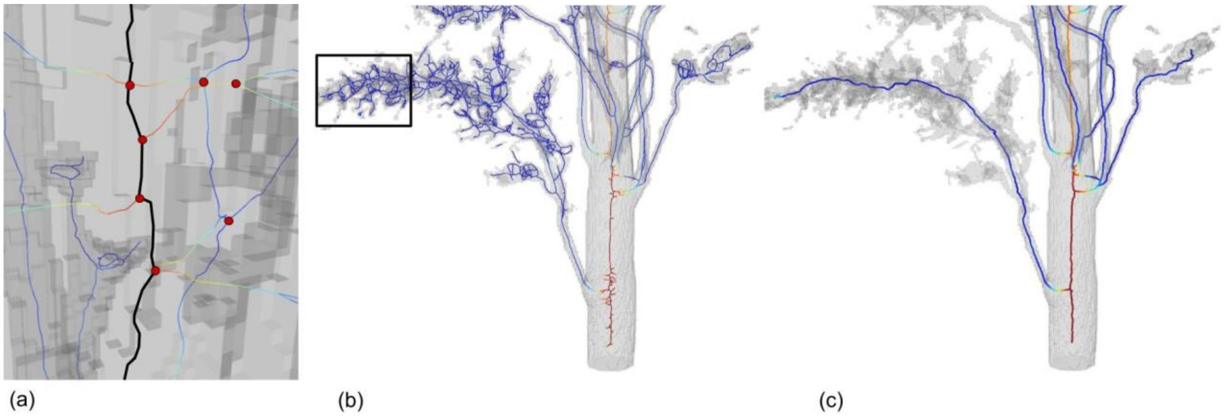

Fig. S7 Primary branches and rachises of representative sorghum panicles. Results shown as detected using our skeleton-based method; rachises are colored in blue and each branch is colored differently. Our method uses geodesic length along the curve skeleton to measure branch and internode lengths, resulting in precise distance measurements regardless of the branching angle or curvature. This allows our method to capture geospatial primary branch traits without any degradation to the genetic signature.

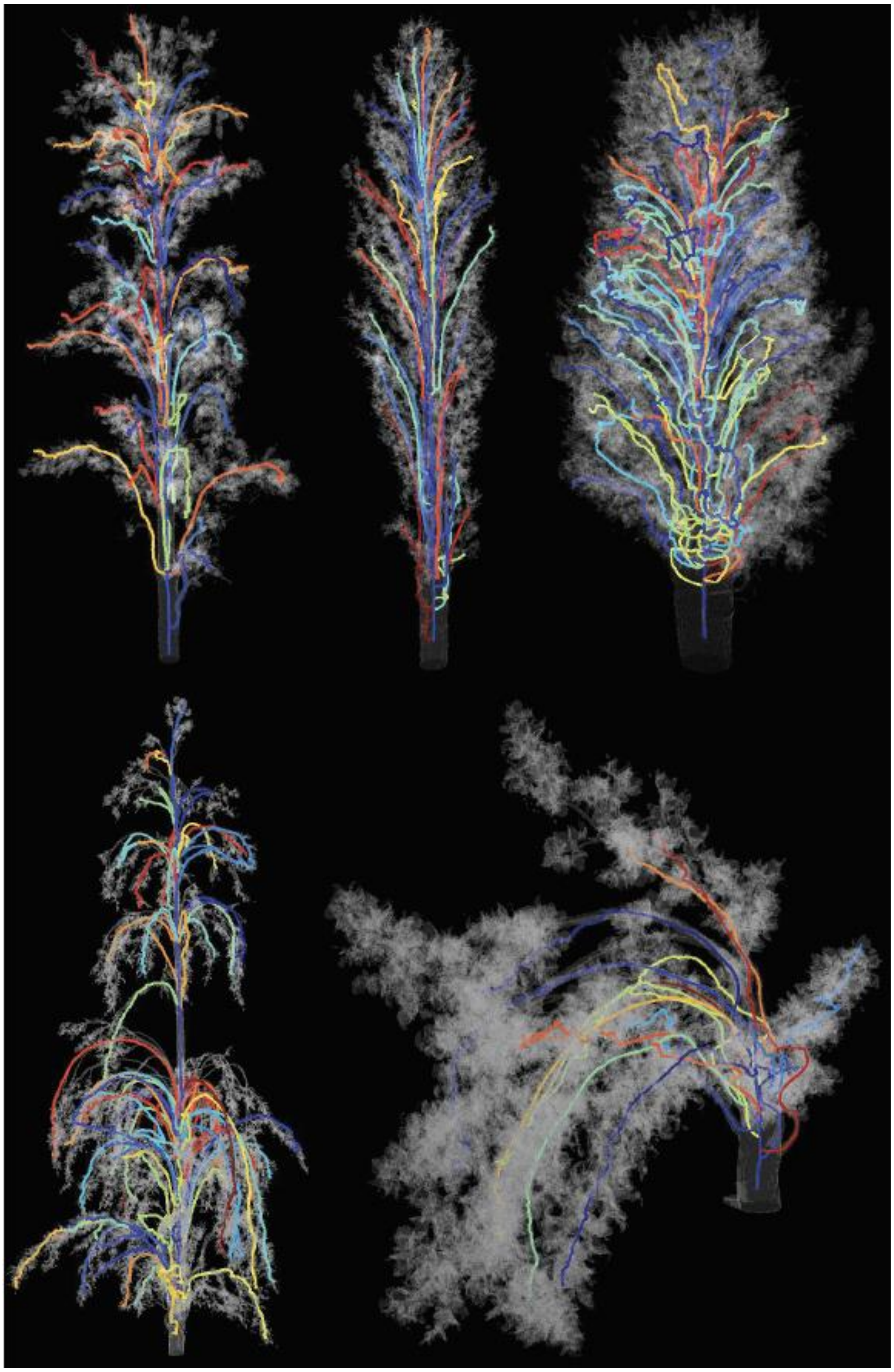

Fig. S8 Trait correlation plot. Spearman's correlation matrix plot ($n = 55$) indicating positive (red) or negative (blue) correlations, with magnitude indicated by circle size. The shadowed squares indicate statistical significance (rho < 0.05).

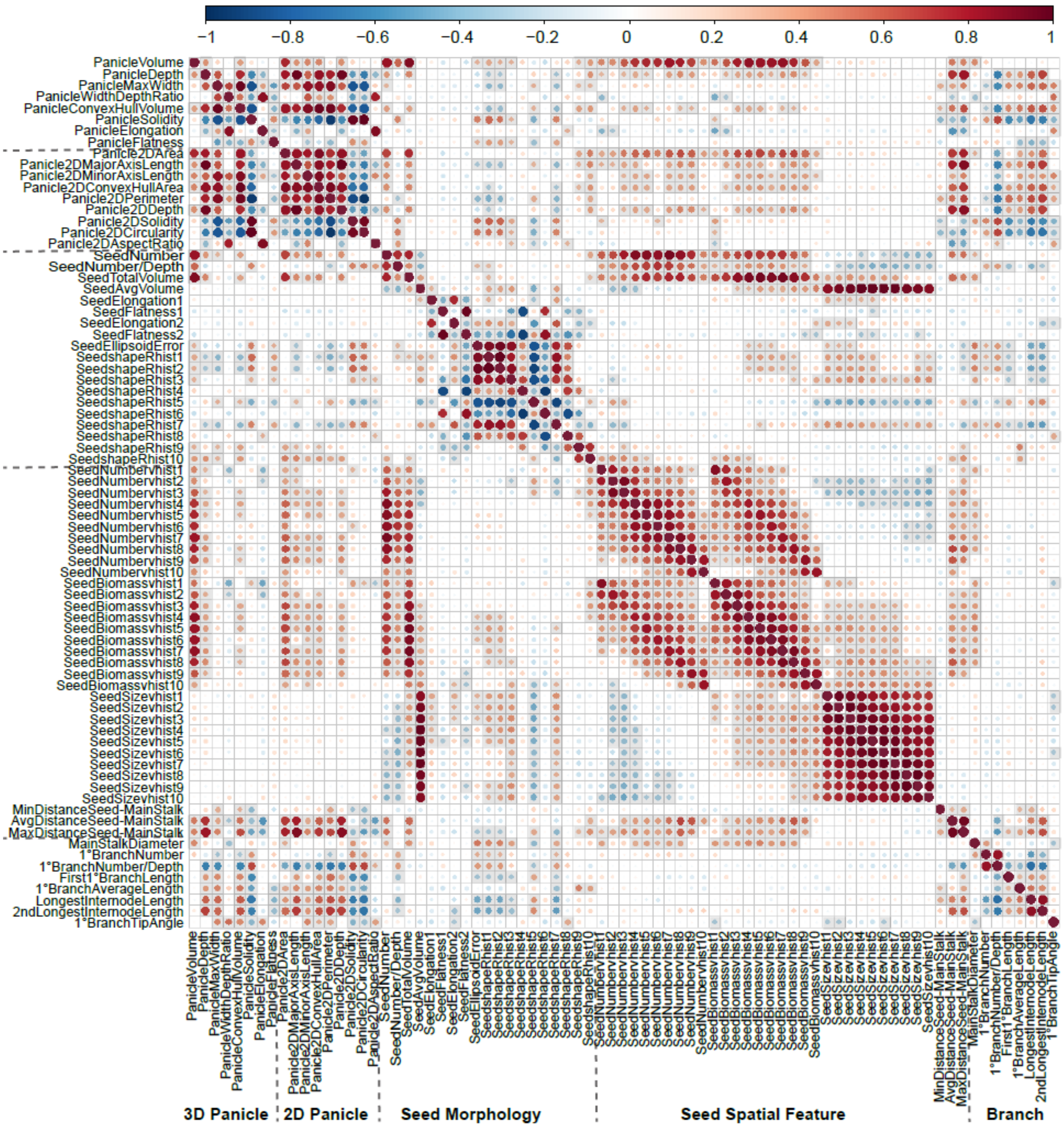

Fig. S9 Distribution and variation for all traits among the five races. For each boxplot, points indicate an outlier beyond the 1st/3rd quartiles ± 1.5 times the interquartile range; the central horizontal line indicates the median; the bottom and top edges of the box represent the 25th and 75th percentiles; and the whiskers extend to the most extreme nonoutlier data point. Each curve in the bottom left plots represents the average value in each bin for each race. The highlighted region shows the range within one standard deviation.

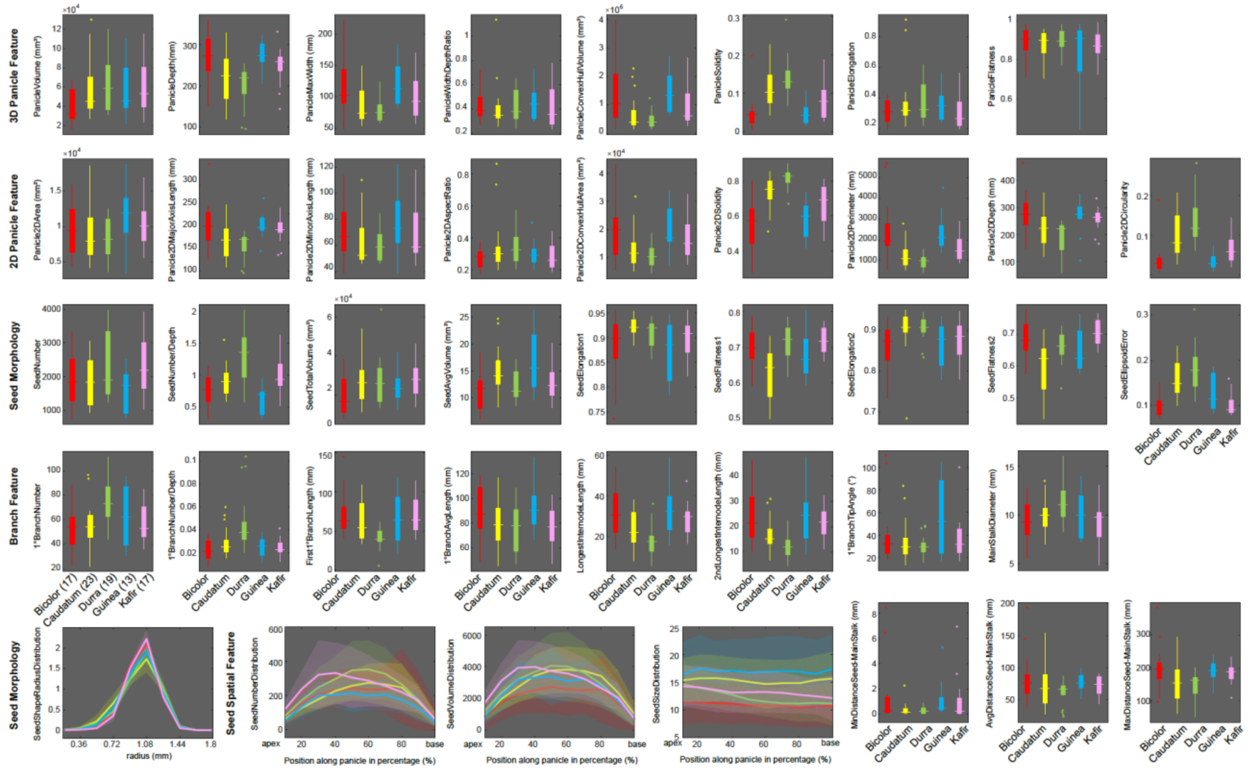

Fig. S10 Principal component analysis and linear discriminant analysis loadings. PCA loadings (left panel) show the contribution of each feature to the variance, with highly positive or highly negative loadings being more important. The PCA-LDA loadings (right panel) show the importance of each feature in terms of its contribution in distinguishing the races.

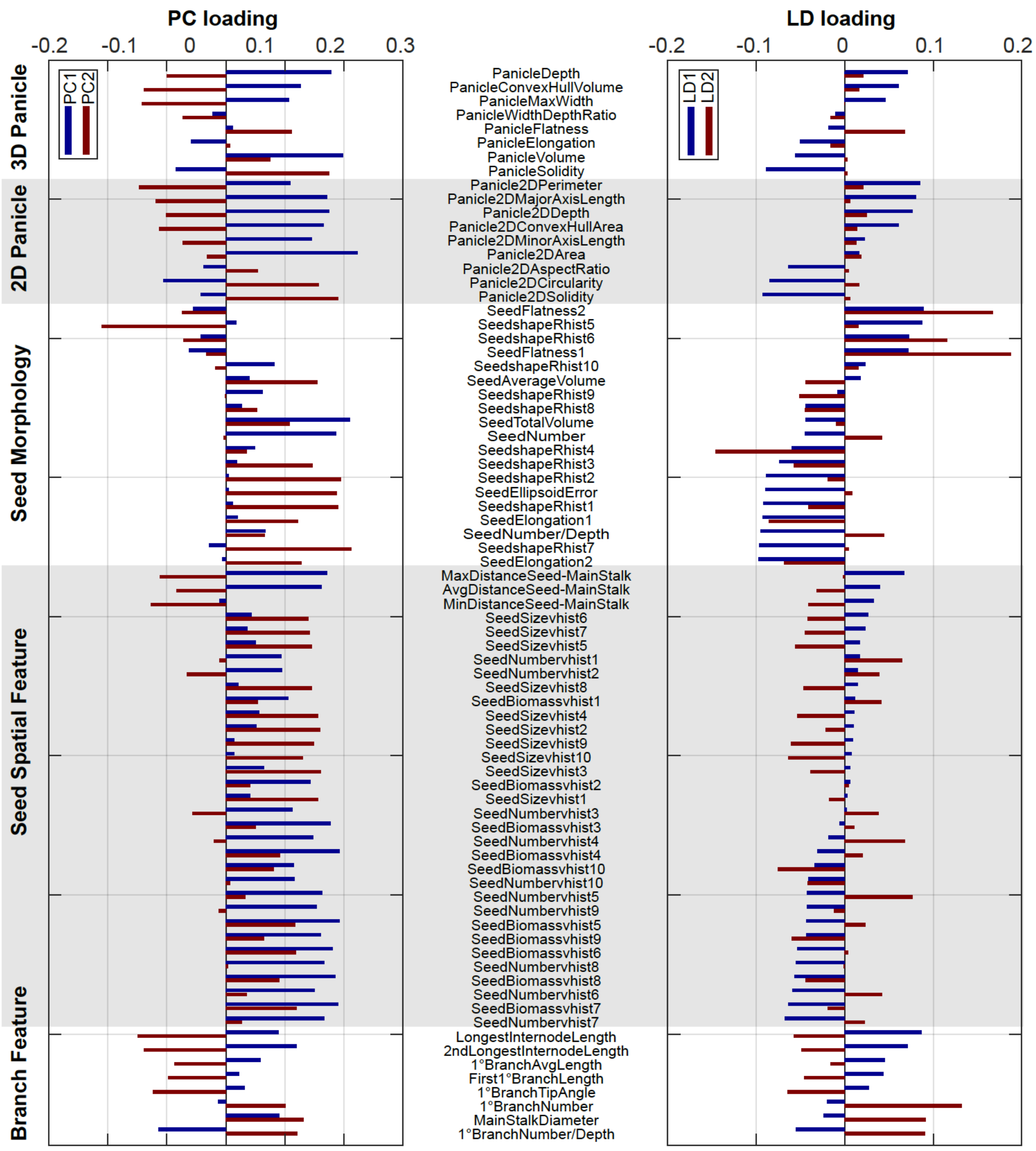

Fig. S11 Isometric feature mapping with all traits. As an alternative to principal components analysis, isometric feature mapping with all traits was performed to examine possible non-linear patterns across multivariate traits. Isomap using the R *vegan* package was performed with varying epsilon values, but this also did not result in distinct separation by botanical race.

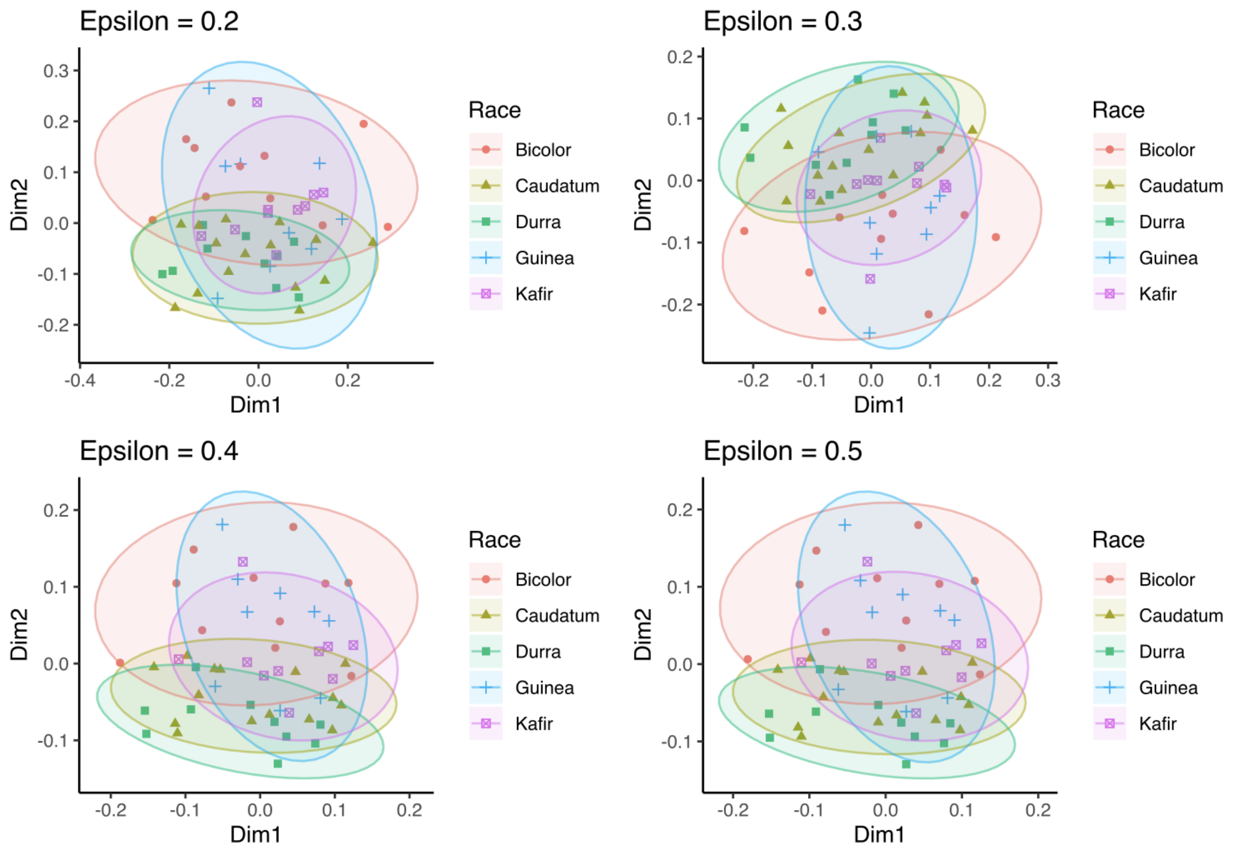

Fig. S12 K-mean clustering. No distinct clustering alternative to botanical race was observed based on panicle phenotype and morphology. (a) Estimated optimal number of clusters for k-means clustering based on average silhouette width. (b) Visualization of k-means clustering results for k = 2, 3, 4, and 5 shows no obvious alternative to the five botanical races based solely on panicle features.

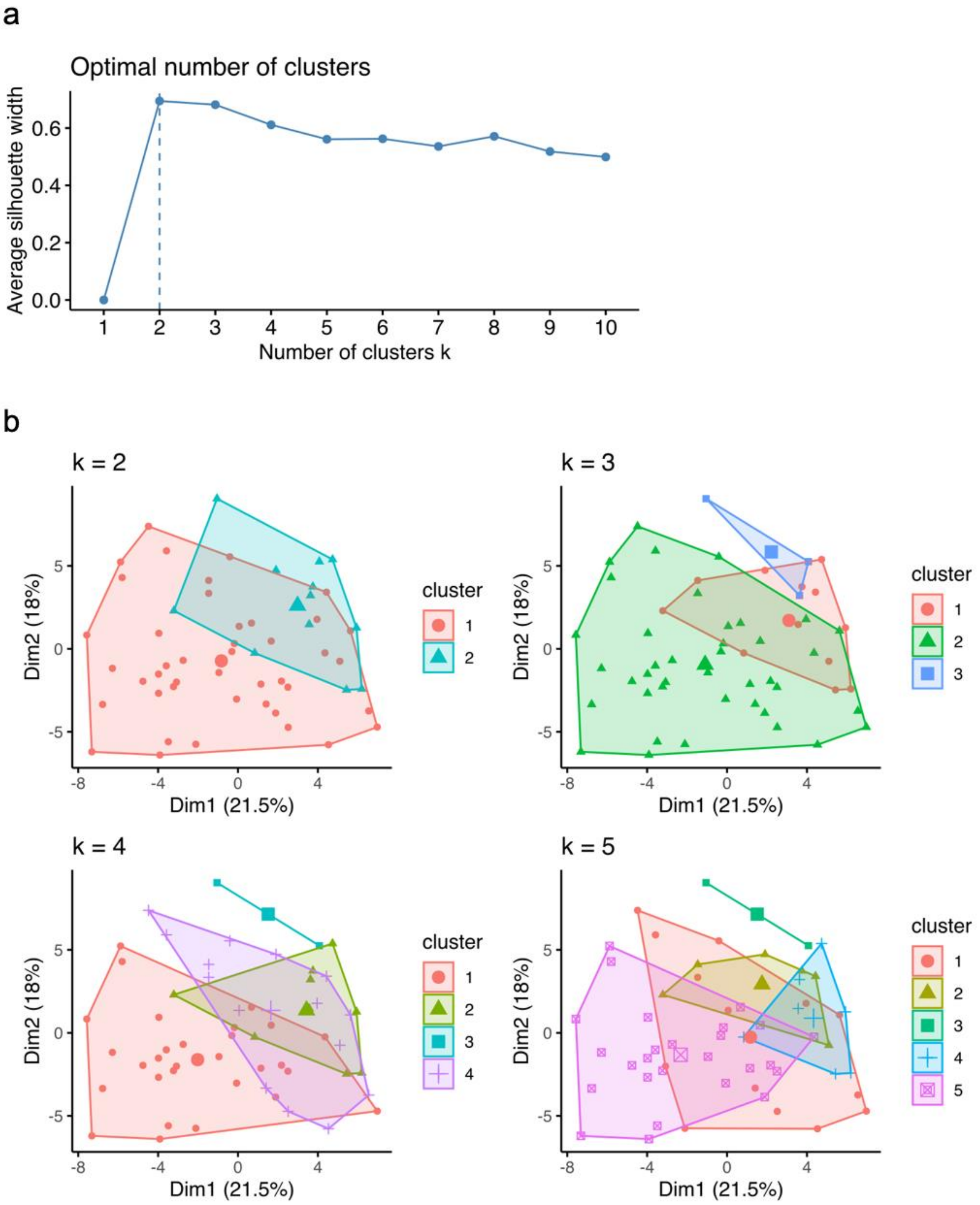

Supplement: Supplementary file 1 — Fig. S1 Examples of sorghum panicle morphology. Fig. S2 Additional details on X‐ray imaging workflow. Fig. S3 Diagrammatic description of seed distribution features. Fig. S4 Thresholding considerations with primary branches. Fig. S5 Stem identification process. Fig. S6 Branch identification process. Fig. S7 Primary branches and rachises of representative sorghum panicles. Fig. S8 Trait correlation plot. Fig. S9 Distribution and variation for all traits among the five races. Fig. S10 Principal component analysis and linear discriminant analysis loadings. Fig. S11 Isometric feature mapping with all traits. Fig. S12 K‐mean clustering. Methods S1 Additional methods on primary branch and rachis trait extraction, skeleton generation, stem identification, primary branch identification and measurements, and manual panicle measurements. [file NPH-226-1873-s001.pdf]
